# Proxy and patient reports of health-related quality of life in a national cancer survey

**DOI:** 10.1186/s12955-017-0823-5

**Published:** 2018-01-05

**Authors:** Jessica K. Roydhouse, Roee Gutman, Nancy L. Keating, Vincent Mor, Ira B. Wilson

**Affiliations:** 1 Department of Health Services, Policy, and Practice, School of Public Health, Brown University, 121 S. Main Street, Providence, RI 02912 USA; 2 Department of Biostatistics, School of Public Health, Brown University, 121 S. Main Street, Providence, RI 02912 USA; 3 Department of Health Care Policy, Harvard Medical School, 180 Longwood Avenue, Boston, MA 02115 USA

**Keywords:** Cancer, Physical health, Mental health, Patient-reported outcomes, Proxy-reported outcomes, Survey

## Abstract

**Background:**

Proxy respondents are frequently used in surveys, including those assessing health-related quality of life (HRQOL). In cancer, most research involving proxies has been undertaken with paired proxy-patient populations, where proxy responses are compared to patient responses for the same individual. In these populations, proxy-patient differences are small and suggest proxy underestimation of patient HRQOL. In practice, however, proxy responses will only be used when patient responses are not available. The difference between proxy and patient reports of patient HRQOL where patients are not able to report for themselves in cancer is not known. The objective of this study was to evaluate the difference between patient and proxy reports of patient HRQOL in a large national cancer survey, and determine if this difference could be mitigated by adjusting for clinical and sociodemographic information about patients.

**Methods:**

Data were from the Cancer Care Outcomes Research and Surveillance (CanCORS) study. Patients or their proxies were recruited within 3–6 months of diagnosis with lung or colorectal cancer. HRQOL was measured using the SF-12 mental and physical composite scales. Differences of ½ SD (=5 points) were considered clinically significant. The primary independent variable was proxy status. Linear regression models were used to adjust for patient sociodemographic and clinical covariates, including cancer stage, patient age and education, and patient co-morbidities.

**Results:**

Of 6471 respondents, 1011 (16%) were proxies. Before adjustment, average proxy-reported scores were lower for both physical (−6.7 points, 95% CI -7.4 to −5.9) and mental (−6 points, 95% CI -6.7 to −5.2) health. Proxy-reported scores remained lower after adjustment (physical: −5.8 points, −6.6 to −5; mental: −5.8 points, −6.6 to 5). Proxy-patient score differences remained clinically and statistically significant, even after adjustment for sociodemographic and clinical variables.

**Conclusions:**

Proxy-reported outcome scores for both physical and mental health were clinically and significantly lower than patient-reported scores for these outcomes. The size of the proxy-patient score differences was not affected by the health domain, and adjustment for sociodemographic and clinical variables had minimal impact.

## Background

Patient-reported outcomes (PROs) are increasingly important in oncology. The Medicare Health Outcomes Survey (MHOS) collects health-related quality of life (HRQOL) and other PRO information from respondents, which is used in the calculation of publicly reported health plan star ratings [1]. Additionally, the National Quality Forum is working to develop PRO performance measures [2]. In oncology, PRO performance measures assessing symptom management processes and outcomes are being actively explored [3, 4].

A major challenge associated with PROs is that patients may be too ill to complete questionnaires. In diseases such as cancer, severely ill patients may comprise a large portion of the study population. Evaluation of the potential for proxy reporters to answer on the patient’s behalf in cancer was primarily conducted using paired studies that include data from proxy-patient dyads [5]. The findings from paired studies evaluating proxy-patient differences and correlations in cancer suggest that proxy-patient differences tend to be small on average and correlations are at least moderate [6–8]. It is not known how well the findings from paired proxy-patient studies will map to unpaired studies, as patients in the latter group are unable to complete questionnaires and thus may differ in important, systematic ways from patients who are able to complete questionnaires. Furthermore, proxies will only be used in practice if patients are unable to complete questionnaires, and thus understanding the differences in this practical context is important.

This question takes on greater importance in light of the many surveys used for public reporting and health policy decision-making that employ proxies to report on behalf of an otherwise missing patient and evaluate the patient’s health status and HRQOL. This includes the Behavioral Risk Factor Surveillance System (BRFSS) [9], MHOS [10, 11], and the National Health Interview Survey (NHIS) [12, 13]. Results from survey populations using proxies to substitute for otherwise missing patients have found that proxies underestimate the prevalence of disease [14, 15] and disability [12], although casemix adjustment may be able to reduce this bias in some cases [13].

To date, few studies in cancer have evaluated the differences between proxy and patient reports in unpaired studies, where proxies are more likely to be needed and used. It is also important to determine whether any differences found can be reduced through adjustment for clinical and sociodemographic characteristics, as such characteristics are frequently collected in surveys. Because HRQOL is an important outcome in cancer, particularly advanced cancer, and a high rate of proxy use can be anticipated due to the nature of disease and treatment, understanding the size of the proxy-patient difference and the potential for mitigating it using routinely collected data is important. We therefore evaluated 1) the size and direction of the difference between proxy and patient reports of patient HRQOL in a large, population-based representative survey of cancer patients; and 2) whether this difference was affected by adjustment for frequently used sociodemographic and clinical covariates. We hypothesized that the use of proxies would be driven by poor patient health, and that proxy HRQOL responses would therefore be consistently lower than patient responses.

## Methods

### Study setting, participants and data sources

The Cancer Care Outcomes Research and Surveillance (CanCORS) study is a large, clinically and demographically representative [16] study of patients with incident lung or colorectal cancer. CanCORS evaluated a number of PROs, including care experience and quality rating [17, 18] and shared decision-making [19–21]. The design and conduct of the study has been reported previously [17, 18, 22]. Patients were enrolled from 2003 to 2005 using rapid case ascertainment from several geographic regions and health systems [18, 19]. Computer-adapted telephone interviewing was used to survey patients, or their proxies if patients were unable to respond or had died, approximately 3 to 6 months post diagnosis. If patients were not able to respond, they were asked if a proxy could answer on their behalf, and to nominate someone who was knowledgeable about their condition and care. Beyond being nominated by a patient, no further eligibility criteria were placed on proxies. Partial, brief or self-administered surveys were also offered if needed.

Sociodemographic information, the presence or absence of co-morbidities, and reports of care experiences, care quality, cancer symptoms, and health-related quality of life were solicited through the computer-adapted telephone interview. Trained abstractors extracted information on patient cancer stage from medical records [18]. If medical records were not available, American Joint Committee on Cancer (AJCC) stage or historical stage (local/regional/distant) was obtained from cancer registries. Questionnaire instruments were based on previously validated or employed instruments [22]. The American Association for Public Opinion Research [16] survey response rate was 51.0% and the cooperation rate was 59.9%. Institutional review boards at all participating institutions approved the study and written or verbal informed consent was obtained depending on the study site.

**Fig. 1:**
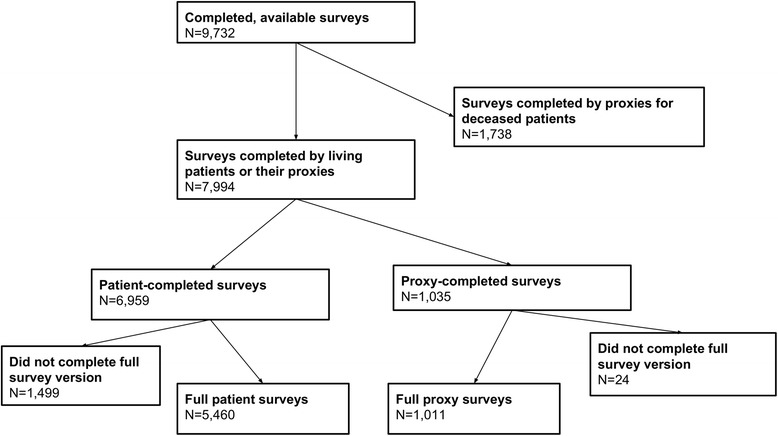
Study Sample Selection Flow Diagram

For this analysis, we restricted the study sample to patients and proxies of living patients who completed the full baseline telephone survey (Fig. 1, *n* = 6471). All patients in our analytic sample were alive at the time of the survey.

### Outcome measures/dependent variables

HRQOL in CanCORS was assessed using questions from the 12-item Short Form (SF-12, version 2) [22], a validated and widely adopted generic HRQOL tool [23]. Proxy and patient questions for the SF-12 were identical, except for patients being asked about “your” health and proxies being asked about “the patient’s” health.

The SF-12 includes 12 questions that cover eight domains: general health, physical function, role-physical, role-emotional, bodily pain, mental health, vitality, and social function (Appendix: Table 4). All but one of the SF-12 items ask respondents to refer to the past 4 weeks when answering. Three and five point scales are used for item scores. Scoring through T-scores using US general population means and standard deviations and weighting produces two composite scales: the physical (PCS) and the mental (MCS). Both scales are calculated using each item, albeit with different weights; thus, missingness in any one item can result in the full scale being missing for that observation. These scales range from 0 (worst) to 100 (best), with normalized standardized T-score means of 50 and standard deviations of 10. Score differences of ½ SD (=5 points) are often treated as clinically significant [24, 25].

### Independent variable and covariates: The adjustment model

In our primary analyses, our independent variable was an indicator for proxy (0/1). The use of an indicator variable for respondent status as part of a regression model is a frequently used approach for accounting for proxy-reported data [11, 26]. We also added several “standard” clinical and sociodemographic covariates, based on the casemix model used for the MHOS, a large national survey of HRQOL that allows for proxy respondents. Because MHOS is not cancer-specific, we also adjusted for cancer type (lung or colorectal) and disease curability (incurable/potentially curable). Incurable disease was defined as AJCC stage IIIB or IV, distant stage, or unstaged for lung cancer and AJCC stage IV, distant stage, or unstaged for colorectal cancer. We included this information as disease stage has been shown to be a predictor of HRQOL in patients with cancer [27, 28].

Our regression models adjusted for several patient sociodemographic characteristics, all of which have been previously shown to predict HRQOL in patients with cancer or cancer survivors: gender [27, 29], marital status [30], race/ethnicity [30], age [29], and educational attainment [31], all of which were solicited during the telephone interview and provided either by the patient, describing themselves (in a patient interview) or the proxy, describing the patient (in a proxy interview). Understanding variation in patient experience by race/ethnicity was a goal of CanCORS [18]. Race/ethnicity was collected using the U.S. Census variables. For this analysis, race/ethnicity was included for consistency with MHOS casemix [32]. We also adjusted for CanCORS study site. The separate models for the two primary outcomes (PCS and MCS) were examined. We also compared adjusted and unadjusted proxy-patient differences for each individual subscale (general health, physical function, role physical, role emotional, bodily pain, mental health, vitality, and social function). Because all subscales are combined in the PCS and MCS calculation, we evaluated the subscales to ensure that no single subscale had significant influence on our results.

Finally, we adjusted for the presence of comorbidities, as comorbidities have also been predictors of HRQOL in patients and survivors [28, 33, 34]. Our analyses adjusted for each of the following patient comorbidities: coronary artery disease (heart attack and/or bypass), heart failure, stroke, arterial bypass, lung disease (asthma, bronchitis, emphysema, or other chronic lung conditions), diabetes/high blood sugar, kidney problem, depression (or other emotional, nervous, or psychiatric problems), previous cancer, and hospitalization within the last year. In patient surveys, these comorbidities were reported by the patient [35], but in proxy surveys the proxy reported whether or not the patient had the comorbidities in question.

Although we felt that it was possible that proxy characteristics could influence their reports of patient HRQOL, most surveys that collect proxy data do not collect proxy characteristics or adjust for them in models. Thus, to be consistent with our goal of examining proxy-patient differences after adjustment for frequently used covariates, we did not include proxy characteristics in our models. However, to provide context we report the most common types of proxy-patient relationships, determined via the proxy questionnaire.

### Statistical analyses

The PCS and MCS were modeled separately for all analyses. Unadjusted patient-proxy differences in mean HRQOL scores were obtained using t-tests. Multivariable linear regression models with all independent variables were used for adjusted analyses. Predicted marginal means were calculated for proxies and patients for each outcome. The assumptions of the linear regression models were evaluated using residuals vs predicted plots, Q-Q plots of residuals, and Cook’s d values. Between-respondent comparisons of covariates were conducted using chi-square tests. All significance tests were performed at the α = 0.05 level, and all analyses were conducted using SAS v9.4.

### Missing data

Multiple imputation (MI) was used for missing data. Missing data in CanCORS were imputed using sequential regression multiple imputation in IVEware [36]. The coefficient of determination (R^2^) was estimated in each of the imputed datasets separately and combined using Harel’s formula [37, 38]. The contribution of variables to the model was evaluated using the multiple partial F-test for MI data [39]. These calculations were performed in R Studio (version 3.2.2). We defined the analytic cohort as respondents with complete covariates. Responses such as “not applicable” or “refused” were treated as incomplete and excluded, resulting in an analytic cohort of *N* = 6426.

For individual items in the SF-12, any non-numeric or “n/a” response is treated as missing, because it cannot be validly summed as part of the composite score. These items are excluded from the calculation, resulting in missing scales for the respondents [40]. Within the analytic cohort, excluded items were infrequent; *N* = 6422/6426 (99.9% of the analytic cohort and 99.2% of the study sample) had valid, numeric responses for all items and the corresponding PCS and MCS scales.

### Sensitivity analyses

We conducted several sensitivity analyses. First, we evaluated the impact of including unstaged patients in our models by estimating the models with these patients excluded. Second, we analyzed the robustness of the results when variables were added or removed from the regression models. For the robustness check, we evaluated the impact of adding wealth (number of months patients could live on their savings), to approximate income; this variable has been used previously in CanCORS analyses [41, 42]. We also evaluated the impact of adding survey timing relative to diagnosis. Next, we evaluated the impact of removing the following variables: 1) all co-morbidities, including hospitalization; 2) hospitalization only.

Third, we implemented our primary models using complete case analysis for missing data rather than the multiply imputed datasets; the complete case analysis approach has been used in several studies evaluating HRQOL in MHOS respondents with cancer [43–45]. Fourth, we evaluated the impact of proxy reports of patient comorbidities on the relationship between comorbidities and HRQOL outcomes in the primary model. Because proxies may report patient conditions such as depression differently than patients [46, 47], we compared the coefficients for all comorbidities in the primary model under three scenarios: 1) with both patient- and proxy-reported data and an indicator variable for proxy status; 2) with both patient- and proxy-reported data and no indicator variable for proxy status; 3) with patient-reported data only. Lastly, as noted previously we also checked that our results were not disproportionately influenced by a single subscale by testing proxy-patient differences at the subscale level.

## Results

Among 6471 participants, 1011 (16%) were proxies. Most proxies were the patient’s spouse/partner (50%) or child (36%). Patients with proxy reporters had similar proportions of the different cancer types but were more likely to have incurable disease (40.6% vs 30.8%), were older, and were less educated (Table 1). Patients with proxy reporters also had more comorbidities and were more likely to have been hospitalized in the preceding year (26.0% vs 19.9%). For comorbidities, the greatest difference was observed for stroke (proxy report: 17.4%, patient report: 8.3%).

**Table 1:** Study sample characteristics: Observed data (*N* = 6471)

Characteristic	Overall N (%)	Proxy-Reported N (%)	Patient-Reported N(%)	*P*-value
Respondent type				
Proxy	1011 (15.6)	1011 (100)	5460 (100)	N/A
Patient	5460 (84.4)			
Patient cancer type				
Colorectal cancer	2907 (44.9)	485 (48.0)	2422 (44.4)	0.0339
Lung cancer	3564 (55.1)	526 (52.0)	3038 (55.6)	
Patient disease stage/curability^a^				
Incurable	2089 (32.3)	410 (40.6)	1679 (30.8)	<0.0001
Potentially curable	4382 (67.7)	601 (59.5)	3781 (69.3)	
CanCORS site				
1	933 (14.4)	65 (6.4)	868 (15.9)	<0.0001
2	1345 (20.8)	206 (20.4)	1139 (20.9)	
3	857 (13.2)	238 (23.5)	619 (11.3)	
4	1359 (21.0)	209 (20.7)	1150 (21.1)	
5	567 (8.8)	75 (7.4)	492 (9.0)	
6	755 (11.7)	124 (12.3)	631 (11.6)	
7	655 (10.1)	94 (9.3)	561 (10.3)	
Patient gender				
Male	3590 (55.5)	680 (67.3)	2910 (53.3)	<0.0001
Female	2881 (44.5)	331 (32.7)	2550 (46.7)	
Patient marital status				
Married/partnered	4003 (61.9)	643 (63.6)	3360 (61.5)	0.1890
Single/divorced/widowed/never married/separated	2433 (37.6)	361 (35.7)	2072 (38.0)	
Missing (blank/refused/unknown)	35 (0.5)	7 (0.7)	28 (0.5)	
Patient race/ethnicity				
White	4400 (68.0)	599 (59.3)	3801 (69.6)	<0.0001
Hispanic	479 (7.4)	108 (10.7)	371 (6.8)	
Black	886 (13.7)	160 (15.8)	726 (13.3)	
Asian (including Native Hawaiian)	370 (5.7)	97 (9.6)	273 (5.0)	
Other (including Native American, multiracial)	322 (5.0)	44 (4.4)	278 (5.1)	
Missing (unknown)	14 (0.2)	3 (0.3)	11 (0.2)	
Patient age				
< =59 years old	1980 (30.6)	131 (13.0)	1849 (33.9)	<0.0001
60–69 years old	1827 (28.2)	237 (23.4)	1590 (29.1)	
70–79 years old	1771 (27.4)	330 (32.6)	1441 (26.4)	
80+ years old	893 (13.8)	313 (31.0)	580 (10.6)	
Patient educational attainment				
< High school (<12th grade completed)	1331 (20.6)	410 (40.6)	921 (16.9)	<0.0001
High school/vocational/some college (<4 years)	3603 (55.7)	445 (44.0)	3158 (57.8)	
College (4 years or degree)/postgraduate	1464 (22.6)	120 (11.9)	1344 (24.6)	
Missing (blank/refused/unknown/not applicable)	73 (1.1)	36 (3.6)	37 (0.7)	
Patient hospitalization in prior year				
No	5035 (77.8)	723 (71.5)	4312 (79.0)	<0.0001
Yes	1348 (20.8)	263 (26.0)	1085 (19.9)	
Missing (blank/refused/unknown/not applicable)	88 (1.4)	25 (2.5)	63 (1.2)	
History of arterial bypass				
No	6106 (94.4)	929 (91.9)	5177 (94.8)	0.0049
Yes	280 (4.3)	60 (5.9)	220 (4.0)	
Missing (blank/refused/unknown/not applicable)	85 (1.3)	22 (2.2)	63 (1.2)	
History of coronary artery disease (heart attack and/or bypass)				
No	5314 (82.1)	772 (76.4)	4542 (83.2)	<0.0001
Yes	1096 (16.9)	228 (22.6)	868 (15.9)	
Missing (blank/refused/unknown/not applicable)	61 (0.9)	11 (1.1)	50 (0.9)	
History of heart failure				
No	5955 (92.0)	875 (86.6)	5080 (93.0)	<0.0001
Yes	408 (6.3)	112 (11.1)	296 (5.4)	
Missing (blank/refused/unknown/not applicable)	108 (1.7)	24 (2.4)	84 (1.5)	
History of stroke				
No	5754 (88.9)	816 (80.7)	4938 (90.4)	<0.0001
Yes	628 (9.7)	176 (17.4)	452 (8.3)	
Missing (blank/refused/unknown/not applicable)	89 (1.4)	19 (1.9)	70 (1.3)	
Chronic lung disease				
No	4906 (75.8)	723 (71.5)	4183 (76.6)	0.0039
Yes	1451 (22.4)	259 (25.6)	1192 (21.8)	
Missing (blank/refused/unknown/not applicable)	114 (1.8)	29 (2.9)	85 (1.6)	
Diabetes				
No	5225 (80.7)	772 (76.4)	4453 (81.6)	0.0004
Yes	1170 (18.1)	221 (21.9)	949 (17.4)	
Missing (blank/refused/unknown/not applicable)	76 (1.2)	18 (1.8)	58 (1.1)	
History of kidney problems				
No	5727 (88.5)	879 (86.9)	4848 (88.8)	0.2488
Yes	650 (10.0)	111 (11.0)	539 (9.9)	
Missing (blank/refused/unknown/not applicable)	94 (1.5)	21 (2.1)	73 (1.3)	
History of depression/psychological problems				
No	4964 (76.7)	749 (74.1)	4215 (77.2)	0.1117
Yes	1421 (22.0)	239 (23.6)	1182 (21.7)	
Missing (blank/refused/unknown/not applicable)	86 (1.3)	23 (2.3)	63 (1.2)	
History of previous cancer				
No	5158 (79.7)	785 (77.7)	4373 (80.1)	0.1068
Yes	1236 (19.1)	211 (20.9)	1025 (18.8)	
Missing (blank/refused/unknown/not applicable)	77 (1.2)	15 (1.5)	62 (1.1)	

^a^For lung cancer, incurable was considered AJCC staging IIIB or above, non-AJCC staging of distant, or unstaged; for CRC, incurable was considered AJCC staging IV, non-AJCC staging of distant, or unstaged

In unadjusted analyses, proxy-reported PCS and MCS scores were clinically and statistically significantly lower. Proxy-reported PCS scores were 6.65 points lower on average (95% CI -7.42 to −5.88). On the PCS, the average proxy-reported score was 33.56 (SE = 0.36), versus an average patient-reported score of 40.21 (SE = 0.15). Proxy-patient differences for the MCS were slightly smaller but still large: average proxy scores were 5.96 points lower (95% CI -6.74 to −5.19). On the MCS, the mean scores were 44.73 (SE = 0.36) and 50.69 (SE = 0.16). For the subscales, proxy-patient differences ranged from −5.4 points (bodily pain) to −8.9 points (physical function); differences of 5 points or greater were seen in either the point estimate or confidence interval of all subscales.

Adjustments using sociodemographic and clinical covariates had minimal effect on proxy coefficient estimates, resulting in clinically and statistically significant proxy-patient conditional differences (Table 2). Similarly, for all subscales, conditional differences of at least 5 points remained after adjustment. Model diagnostics did not indicate severe violations of the multivariate linear regression assumptions.

**Table 2:** Proxy-patient differences for health-related quality of life outcomes

Model type	Physical Composite Scale Score					Mental Composite Scale Score				
	N	R^2^	95% CI	Coefficient	95% CI	N	R^2^	95% CI	Coefficient	95% CI
Unadjusted (proxy variable as sole covariate)	6422	0.04	0.04–0.05	−6.65	−7.42 to −5.88	6422	0.03	0.03–0.04	−5.96	−6.74 to −5.19
Full casemix model, minus comorbidities and recent hospitalization	6422	0.12	0.12–0.13	−6.32	−7.13 to −5.53	6422	0.09	0.08–0.09	−6.54	−7.36 to −5.72
Full casemix model with comorbidities, minus recent hospitalization	6422	0.16	0.15–0.18	−5.83	−6.61 to −5.04	6422	0.16	0.15–0.17	−5.85	−6.64 to −5.05
Full casemix model	6422	0.17	0.16–0.18	−5.79	−6.57 to −5.00	6422	0.16	0.15–0.17	−5.83	−6.62 to −5.03
Full casemix model, without unstaged patients^a^	6087	0.17	0.16–0.18	−5.62	−6.43 to −4.81	6087	0.16	0.15–0.17	−5.54	−6.36 to −4.73
Full casemix model plus wealth	6422	0.18	0.17–0.19	−5.72	−6.51 to −4.94	6422	0.17	0.16–0.18	−5.77	−6.56 to −4.98
Full casemix model plus survey timing^b^	6420	0.17	0.16–0.18	−5.92	−6.71 to −5.13	6420	0.16	0.15–0.17	−5.87	−6.67 to −5.07

^a^*N* = 335 individuals in the analytic cohort were unstaged; ^b^*N* = 2 individuals had missing survey timing information and therefore *N* = 6420 were included in this analysis

In sensitivity analyses (Table 2), excluding unstaged patients had only small effects on the average proxy scores, with changes of 0.10–0.30 points depending on the outcome. Adjusting for wealth did not substantially reduce the gap between proxy and patient scores for both PCS and MCS models, although this variable was statistically different from zero for both outcomes (F < 0.05 for both). With wealth included, proxy-patient conditional differences for both outcomes remained clinically and statistically different: proxy PCS scores were 5.72 points lower on average (95% CI -6.51 to −4.94) and proxy MCS scores were 5.77 points lower on average (95% CI -6.56 to −4.98). Similarly, adjusting for survey timing had a minimal effect on conditional proxy-patient differences. The coefficient for survey timing was statistically different from zero for PCS (F < 0.05) but not for MCS (F > 0.05). In both cases, the point estimate for the average conditional difference changed only slightly; the proxy-patient conditional difference increased by 0.13 points for PCS and 0.04 points for MCS.

Sensitivity analyses that excluded comorbidities and hospitalization (Table 3) showed that models including these covariates were significantly more effective in predicting both outcomes (F-tests of *p* < 0.05 for both outcomes for these analyses). Excluding co-morbidities and hospitalization exacerbated proxy-patient differences for both PCS and MCS outcomes; proxy-patient conditional differences increased by 0.53 points for PCS and 0.71 points for MCS. Including co-morbidities but not hospitalization resulted in an increased proxy-patient conditional difference of 0.04 points for PCS and 0.02 points for MCS.

**Table 3:** Association of comorbidities and hospitalization with HRQOL outcomes: impact of proxy data

Co-morbidity & hospitalization coefficients	Physical Composite Scale Score			Mental Composite Scale Score		
	Proxy data & coefficient	Proxy data	No proxy data	Proxy data & coefficient	Proxy data	No proxy data
	*N* = 6422 *R*^2^ = 0.17 (0.16–0.18)	*N* = 6422 *R*^2^ = 0.14 (0.13–0.15)	*N* = 5425 *R*^2^ = 0.14 (0.13–0.15)	*N* = 6422 *R*^2^ = 0.16 (0.15–0.17)	*N* = 6422 *R*^2^ = 0.13 (0.12–0.14)	*N* = 5425 *R*^2^ = 0.13 (0.12–0.14)
Hospitalized in prior year (vs not)	−2.14 (−2.81 to −1.47)	−2.21 (−2.89 to −1.52)	−1.96 (−2.70 to −1.23)	−1.03 (−1.70 to −0.35)	−1.10 (−1.78 to −0.42)	−1.41 (−2.13 to −0.70)
History of coronary artery disease (vs no history)	−0.55 (−1.32 to 0.22)	−0.45 (−1.24 to 0.33)	−0.64 (−1.50 to 0.22)	−0.68 (−1.46 to 0.09)	−0.59 (−1.38 to 0.20)	−0.62 (−1.46 to 0.22)
History of heart failure (vs no history)	−3.49 (−4.63 to −2.36)	−3.88 (−5.04 to −2.73)	−3.71 (−5.03 to −2.39)	−0.25 (−1.43 to 0.93)	−0.64 (−1.83 to 0.55)	−0.72 (−2.05 to 0.60)
History of stroke (vs no history)	−1.74 (−2.64 to −0.85)	−2.22 (−3.13 to −1.32)	−1.72 (−2.77 to −0.68)	0.16 (−0.74 to 1.07)	−0.32 (−1.24 to 0.60)	−0.24 (−1.27 to 0.78)
History of arterial bypass (vs no history)	−0.99 (−2.29 to 0.32)	−1.05 (−2.38 to 0.27)	−1.92 (−3.37 to −0.47)	−1.90 (−3.23 to −0.57)	−1.97 (−3.32 to −0.63)	−1.51 (−2.94 to −0.08)
Has chronic lung disease (vs doesn’t have)	−3.52 (−4.19 to −2.85)	−3.62 (−4.29 to −2.94)	−3.80 (−4.54 to −3.06)	−0.46 (−1.13 to 0.20)	−0.56 (−1.24 to 0.12)	−0.34 (−1.05 to 0.37)
Has diabetes (vs doesn’t have)	−1.23 (−1.93 to −0.52)	−1.16 (−1.88 to −0.45)	−1.45 (−2.23 to −0.67)	−0.80 (−1.51 to −0.10)	−0.74 (−1.46 to −0.03)	−0.86 (−1.61 to −0.10)
History of kidney problem (vs no history)	−1.59 (−2.47 to −0.72)	−1.55 (−2.44 to −0.66)	−1.87 (−2.83 to −0.91)	−0.09 (−0.98 to 0.80)	−0.04 (−0.94 to 0.86)	0.14 (−0.80 to 1.08)
History of depression/ psychological problems (vs none)	−0.47 (−1.12 to 0.18)	−0.77 (−1.43 to −0.11)	−0.74 (−1.46 to −0.03)	−7.38 (−8.05 to −6.72)	−7.68 (−8.36 to −7.01)	−7.26 (−7.95 to −6.56)
History of previous cancer diagnosis (vs no history)	−0.51 (−1.20 to 0.18)	−0.45 (−1.15 to 0.25)	−0.54 (−1.30 to 0.21)	−0.19 (−0.88 to 0.50)	−0.13 (−0.83 to 0.57)	0.04 (−0.70 to 0.77)

The results from the primary analysis were similar when complete case analysis rather than analysis of multiply imputed data was used (data not shown). Finally, the associations between comorbidities and HRQOL scores were similar whether or not proxy data were included (Table 3).

## Discussion

In a large national survey of cancer patients in which proxy reports were used to substitute for unavailable patient reports, proxy and patient reports of patient HRQOL had large, clinically relevant differences. Proxy-reported scores were significantly lower than patient scores, indicating worse HRQOL. Furthermore, these differences persisted even after adjustment for clinical and sociodemographic covariates, and changes to the covariates that were included in model had minimal effects. These findings were also robust to different approaches for addressing missing data.

In contrast to previous paired proxy-patient studies in cancer that found only small differences in proxy and patient-reported HRQOL, we found that differences between patient and proxy reports of patient HRQOL were relatively large. For example, Tang and McCorkle’s review of proxy-patient concordance studies in terminally-ill cancer patients found that most studies had small mean differences for physical HRQOL dimensions, with moderate differences seen for more subjective HRQOL aspects such as fatigue and emotional function [6]. Similarly, Sneeuw and colleagues’ review of proxy-patient dyad studies in a range of disease groups, including cancer, found generally small differences between proxy-patient pairs, and only saw more extreme differences in studies with small sample sizes [7]. However, in a study with a large sample size we found relatively large and clinically important differences for both mental and physical dimensions of HRQOL. This suggests that proxies in our study may represent a sicker population. Patients who were too ill to participate in a lengthy interview may have requested that a proxy complete the interview on their behalf. It is possible that the covariates that were collected in the CanCORS survey and used in our model did not adequately capture this decision. Additional information, such as the reason for non-response and the rationale for nominating a specific person as a proxy, may have been helpful and could potentially be considered in future studies. Another possibility is that this difference may be due to proxy bias. Schwarz and Wellens suggest that proxy reporters use different sources of information when making reports compared to individuals making a self-report [48]; in this vein, Snow and colleagues note that because patients know more about themselves than proxies do, proxy bias should be larger for less observable constructs [49]. In our study, however, we found that proxy-patient differences were similar for both physical and mental health. Future qualitative research that investigates the processes and decision-making involved in proxy reporting may be beneficial in addressing some of these issues.

Our findings of similar levels of proxy-patient differences for physical and mental health outcomes are not completely consistent with evaluations of proxy response bias among elderly Medicare patients. Using the Medicare Current Beneficiary Survey, Li and colleagues found higher levels of proxy-patient difference for less observable domains, such as cognitive abilities, and lower levels for more observable domains such as mobility, with no significant differences found for highly observable domains such as seeing or eating solid foods [50]. These differences were obtained from a propensity-matched analysis, accounting for sociodemographic variables such as age and gender as well as clinical information such as the Charlson Comorbidity Index. Our analysis did not use propensity score matching, but adjusted for several co-morbidities as well as for information about disease type and stage as well as socio-demographics in a regression model. One possible explanation is that these co-morbidities may be less important in predicting health status for patients with cancer; it is likely that cancer, rather than, for example, a history of heart failure, is the more proximal driver of poorer health status. Another possibility is that the choice of measurement tool may be a factor. For example, Ellis and colleagues assessed patient-proxy differences among MHOS respondents using the SF-36, and found unadjusted patient scores to be approximately 7 points higher than unadjusted proxy scores for both the PCS and MCS [10]. These differences are consistent with our findings, although relatively few of the respondents in Ellis et al.’s analysis had cancer.

Although previous studies have found discrepancies between proxy and patient reports of comorbidities such as depression [46], the proportion of patients with depression was similar for both proxy- and patient-reported data in our analyses. Furthermore, in our data, the impact of comorbidities on HRQOL was the same regardless of whether or not proxy-reported data were included. One explanation for this may be that while proxy-reported rates of comorbidities were higher, the distribution of comorbidities was roughly similar between respondent types for most of the included comorbidities. Additionally, with the exception of depression in the model for the mental health composite outcome, the average difference in HRQOL between respondents with and without a given comorbidity was relatively small and not clinically significant (<5 points). Alternately, our measure of comorbidity, which assessed the presence of comorbidity but not its severity, may have been insufficiently sensitive to capture comorbidities that were severe enough to impact HRQOL.

Conclusions regarding the impact of proxy reporting in surveys have varied, possibly due to the different outcomes for which proxy respondents have been employed. Some analyses have found the impact of including proxy reports in surveys to be minimal [51] so adjustment can be effective in minimizing proxy-introduced impact [13], but others found that further information about proxies is required for better adjustment [12, 52]. With regard to surveys of HRQOL in cancer patients, our findings indicate that proxy reports differ significantly from patient reports, and regression adjustment using sociodemographic and clinical covariates has at minimal impact on this difference. It is possible that identifying and including additional covariates predictive of patient illness may reduce these differences, particularly because the fully adjusted models only explained <20% of the variance of both outcomes. However, even models that include symptoms as predictors of HRQOL result in less than 30% of the variance in outcomes explained [53]. Similar levels of explanatory power are reported for paired studies evaluating factors associated with proxy-patient concordance [54].

This study had several limitations. First, the CanCORS data, while population-based and nationally representative, were collected several years ago. However, there is no reason that the models would not be valid when evaluating a methodological issue such as evaluating proxy-patient differences in HRQOL. Second, the data used in this paper are cross-sectional; however, many population-based surveys and most studies evaluating patient-proxy concordance in HRQOL employ cross-sectional designs [5]. Third, the CanCORS response rate was 51%; while not ideal, this rate is similar to the response rates for other large population-based national surveys such as the BRFSS [55] that are used to inform health policy and practice. Finally, although using ½ SD as a marker of clinical or minimally important difference is common, it is not the only metric for estimating a minimally important difference. Differences of two to three points on the SF-12 have been considered minimally important in studies with patients with prostate cancer [56]. In a study with patients with extramedullary spinal tumors, score differences of 2.8 points were proposed as minimally important for the PCS and differences of 10.7 for the MCS [57]. Since minimal differences may vary by population and context [58], the generalizability of these varying thresholds to our study, which included patients with lung and colorectal tumors, is not clear. We did not identify a clearly established minimal difference threshold for our survey population and context in the literature. Nonetheless, in our study we identified large and persistent differences that were only minimally affected by adjustment for sociodemographic and clinical covariates.

## Conclusions

In summary, proxy reports of patient HRQOL that are used to substitute for otherwise missing patient reports are clinically and statistically different from available patient reports. Adjustments using frequently employed sociodemographic, clinical and comorbidity covariates have a minimal effect on these differences. In situations of high rates of proxy use, the effect of proxies can be consequential, particularly if the results of such estimates will be employed in performance measures or used to inform policy decisions.

## Appendix

**Table 4:** Items and Scoring for the SF-12 (v2)^a^

Item number	Item content	Time period	Score range	Sub-scale
1	General rating of health	Now	1–5	General health
2	Limitations in moderate activities due to health	Now	1–3	Physical function
3	Limited in climbing stairs due to health	Now	1–3	Physical function
4	Accomplished less due to physical health	Past 4 weeks	1–5	Role physical
5	Limited in daily activities due to physical health	Past 4 weeks	1–5	Role physical
6	Accomplished less due to emotional problems	Past 4 weeks	1–5	Role emotional
7	Less careful due to emotional problems	Past 4 weeks	1–5	Role emotional
8	Pain affected normal work	Past 4 weeks	1–5	Bodily pain
9	Felt calm and peaceful	Past 4 weeks	1–5	Mental health
10	Had a lot of energy	Past 4 weeks	1–5	Vitality
11	Felt down and depressed	Past 4 weeks	1–5	Mental health
12	Physical health or emotional problems affected social activities	Past 4 weeks	1–5	Social function

^a^All items used in calculation of each composite scale, however with differing weights

## Abbreviations

AJCC: American Joint Committee on Cancer
BRFSS: Behavioral Risk Factor Surveillance System
CanCORS: Cancer Care Outcomes Research and Surveillance
HRQOL: Health-related quality of life
MCS: Mental Component Summary (also called the Mental Health Composite Scale)
MHOS: Medicare Health Outcomes Survey
MI: Multiple imputation
NHIS: National Health Interview Survey
PCS: Physical Component Summary (also called the Physical Health Composite Scale)
PROs: Patient-Reported Outcomes
SF-12: Short Form-12
